# Diversity of Microfungi in a High Radon Cave Ecosystem

**DOI:** 10.3389/fmicb.2022.869661

**Published:** 2022-04-27

**Authors:** Tamara Martin-Pozas, Alena Nováková, Valme Jurado, Angel Fernandez-Cortes, Soledad Cuezva, Cesareo Saiz-Jimenez, Sergio Sanchez-Moral

**Affiliations:** ^1^Department of Geology, National Museum of Natural Sciences (MNCN-CSIC), Madrid, Spain; ^2^Laboratory of Fungal Genetics and Metabolism, Institute of Microbiology of the CAS, Prague, Czechia; ^3^Department of Agrochemistry, Environmental Microbiology and Soil Conservation, Institute of Natural Resources and Agricultural Biology (IRNAS-CSIC), Seville, Spain; ^4^Department of Biology and Geology, University of Almeria, Almeria, Spain; ^5^Department of Geology, Geography and Environment, University of Alcala, Alcala de Henares, Spain

**Keywords:** fungal outbreak, Castañar Cave, radon, ionizing radiation, *Ascomycota*, *Basidiomycota*

## Abstract

Castañar Cave is a clear example of an oligotrophic ecosystem with high hygrothermal stability both seasonal and interannual and the particularity of registering extraordinary levels of environmental radiation. These environmental conditions make the cave an ideal laboratory to evaluate both the responses of the subterranean environment to sudden changes in the matter and energy fluxes with the exterior and also any impact derived from its use as a tourist resource under a very restrictive access regime. In 2008, a fungal outbreak provoked by a vomit contaminated the sediments which were removed and subsequently treated with hydrogen peroxide. Fungal surveys were carried out in 2008 and 2009. The visits were resumed in 2014. Here, 12 years after the outbreak, we present an exhaustive study on the cave sediments in order to know the distribution of the different fungal taxa, as well as the prevalence and spatio-temporal evolution of the fungi caused by the vomit over the years under the conditions of relative isolation and high radiation that characterize this cave.

## Introduction

Castañar Cave (Castañar de Ibor, Caceres, Spain) was discovered in 1967, declared Natural Monument in 1997 and opened to visits in 2003. The interest of this cavity lies in its great variety of speleothems formed mainly by aragonite and calcite of low magnesium (Alonso-Zarza et al., 2011) and in its very high concentrations of radon (^222^Rn) in air with annual average above 30 kBq/m^3^ (Lario et al., 2006; Garcia-Guinea et al., 2013). These values are much higher than the average ^222^Rn concentrations found in most caves studied around the world (0.5–8.3 kBq/m^3^; Cigna, 2005; Somlai et al., 2011). ^222^Rn was used as tracer for assessing ventilation of Castañar Cave because its variations depend only on the exchange of air with the exterior (Fernandez-Cortes et al., 2009). The slates hosting the cave contain uranium (39.2 ± 5.2 mg.kg^−1^; Garcia-Guinea et al., 2013), so that its disintegration to radon and the subsequently ^222^Rn exhalation from bedrock entails a continuous source of this gas to cave atmosphere. The weathering leakage processes of the bedrock also favor the remobilization of radionuclides *via* leaching and their later settlement into the cave environment associated to mineral phases of cave deposits (Garcia-Guinea et al., 2013). This favors maintaining the high ^222^Rn activity of cave air since there is a continuous regeneration of this gas due to the long-lived of these radionuclides of the radium radioactive decay chain.

The morning of August 26, 2008, on the cave walls and ground sediments appeared long, white fungal mycelia as the result of the vomit of a visitor, 40 h before. The vomit area was located at 17 m from the cave entrance and the ground sediments exhibited a widespread fungal colonization after the disturbance. In the following days, visitors stepped on the sediments colonized by the fungi, and detritus, mycelia and spores were distributed along the touristic trail, as evidenced by the presence of patches of fungal growth farther than the vomit area (Jurado et al., 2010). To help to control the outbreak, the entire area directly affected by vomiting was cleaned and subsequently treated with hydrogen peroxide and the visits were cancelled on September 11, 2008, 16 days after the spillage. In 2009, the cave was only visited by scientists and staff people for controlling the fungal outbreak. The cave was reopened in 2014 but the number of visitors/year was reduced from 1,500 to 1,600 in 2008, to an annual maximum of 450 visitors. Besides, since the cave re-opened, in order to minimize the entrance of external material, the visitors uses clean and uncontaminated suits and shoe covers.

In Castañar Cave, we had the opportunity to carry out extensive microbiological studies on 2008 and 2009, up to 12 months after the fungal outbreak, and again 12 years after the outbreak in a subsequent and exhaustive study carried out in 2020. Castañar Cave is a natural environment with high levels of ionizing radiation but also a low energy cave and high environmental stability throughout the annual cycle (Fernandez-Cortes et al., 2011) which make it more sensitive to the entry of matter and energy from outside. There are a few reports about the dominance of microbial communities in radioactive environments such as the International Space Station and Chernobyl reactor (Dadachova and Casadevall, 2008; Van Houdt et al., 2012; Romsdahl et al., 2018). These works give some examples of microbial species and melanized fungi with a high tolerance to radioactivity. However, research works in natural environments with high ionizing radiation and on the evolution of microbial populations in these environments are very scarce (Siasou et al., 2017).

The direct control that environmental conditions exert on the distribution of fungi in subterranean ecosystems has recently been demonstrated (Jurado et al., 2021; Sanchez-Moral et al., 2021). The objective of this study is to know the distribution of the different fungal taxa throughout Castañar Cave, as well as the prevalence and spatio-temporal evolution of the fungi caused by the vomit over the years under conditions of high isolation of the cave atmosphere from the outside and an outstanding radon activity.

## Materials and Methods

### Site

Castañar Cave (SW Spain, 39°37′40´´N, 5°24′59´´W, 590 m.a.s.l.) is currently located under a natural olive grove that grows in a temperate and semi-arid climate with a rainfall regime of less than 500 mm per year, long periods of drought in summer and maximum rains in autumn. Outside, the annual average temperature is 15.9°C and the average relative humidity is 62.5%.

**Figure 1:**
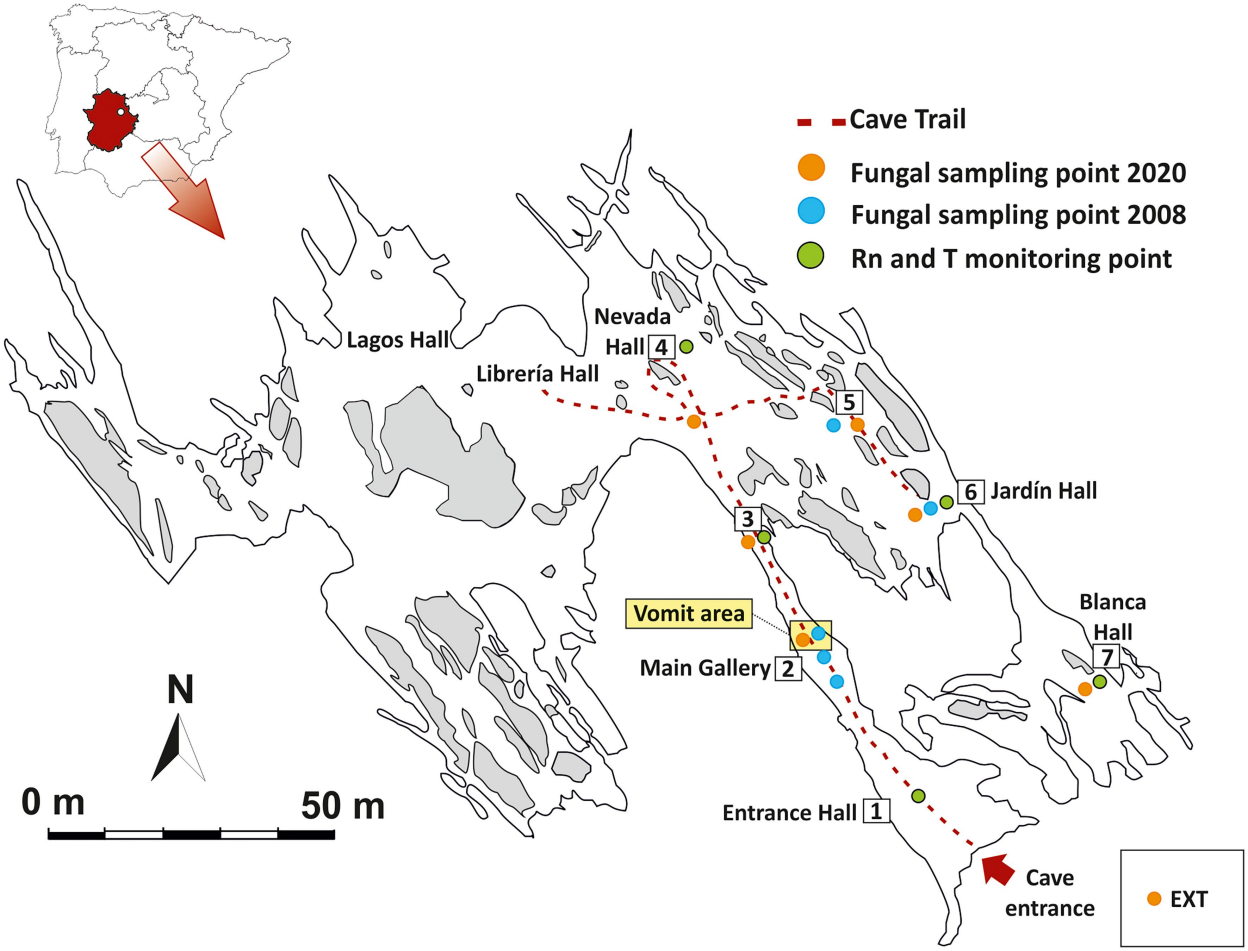
Map of Castañar Cave with location of sampling points, passive radon dosimeters and thermohygrometer recorders for environmental monitoring. EXT: Exterior.

The cave was developed by dissolution of dolomite strata interbedded in shales and greywackes of the Precambrian Age (Alonso-Zarza and Martin-Perez, 2008). Castañar Cave is a small karstic cavity with a cumulative length of 2,135 m, distributed in six main and wider halls (e.g., Sala Nevada) and some narrow galleries connecting them, with heights of less than 3 m in any case (Figure 1). The cave entrance is a unique vertical access, 9 m long over an area of 1.5 m^2^, with a quasi-hermetic door installed at the entrance. All these characteristics make Castañar a low energy cave with very high ^222^Rn gas concentration and high environmental stability throughout the annual cycle (Fernandez-Cortes et al., 2011).

### Environmental Surveys

Variations in ^222^Rn concentration and air temperature are two key environmental parameters to determine the exchange rates of matter and energy and the main connection pathways between the exterior and the cavity. A set of five autonomous thermohygrometer recorders (Tinytag TGP-4500) and five nuclear track-etch passive detectors equipped with a solid state LR-115 type film (Kodak) were installed inside the cave, distributed according to the geomorphological characteristics of the subterranean galleries and the distance to the entrance (Figure 1). This monitoring network makes it possible to obtain monthly mean values of the ^222^Rn concentration of the subterranean air and a continuous thermo-hygrometric record at each point.

### Microbiological Analysis

On December 3, 2020, seven sediment samples (approximately 3–4 cm depth) were collected in Castañar Cave (P1-P7) and one from the exterior soil (E) near the cave entrance (Figure 1). All samples were collected using sterile spatulas and immediately suspended in DNA/RNA Shield™ into sterile polypropylene tubes. Then, the samples were stored at 4°C, in the dark during transportation and were kept at −80°C until processing.

Total DNA was extracted from environmental samples using the QIAGEN Power Soil Kit, following the manufacturer’s instructions. Two independent DNA extractions were carried out for each sampling point, except in P1 where we only collected one sample. Extracted DNA was used as template for generating PCR-amplicons. The internal transcribed spacer 2 (ITS2) of the nuclear ribosomal DNA (about 400 bp) was amplified using the primers ITS86F (5 ‘GTGAATCATCGAATCTTTGAA 3′; Turenne et al., 1999) and ITS4 (5 ‘TCCTCCGCTTATTGATATGC 3′; White et al., 1990). Amplifications settings were as follows: 95°C for 5 min followed by 35 cycles consisting of 95°C for 30s, 49°C for 30 s, and 72°C for 30s and a 10-min elongation step at 72°C. PCR-amplicons were sequenced by high-throughput sequencing at AllGenetics Company (A Coruña, Spain) on the Illumina MiSeq platform using the paired-end reads 2 × 300 bp reagent kit, according to the manufacturer’s instructions. Extraction and PCR blanks were used.

To analyze fungal community composition, Raw fastq files were processed with QIIME2 (Bolyen et al., 2019). Briefly, DADA2 (Callahan et al., 2016) filtered the raw data according to the quality, generating an amplicon sequence variant (ASV) table. Then, taxonomic assignments were determined for ASVs using qiime2-feature-classifier classify-sklearn against the UNITE fungal ITS database (version 8.0; UNITE Community, 2019) and trained with Naive Bayes classifier on the full reference sequences. Taxa were also blasted (BlastN) against GenBank for fungal verification. All fungal names were reported according to the MycoBank Database. The sequencing data was further imported into the R environment 3.6.0 and processed using the phyloseq package (McMurdie and Holmes, 2013). Barplots and clustered heat maps by euclidean distance were prepared using ggplot and pheatmap packages to illustrate the composition of fungal communities in Castañar Cave. Alpha diversity was calculated using Shannon diversity index and Faith’s Phylogenetic Diversity, directly using QIIME2. The raw reads were deposited into the NCBI Sequence Read Archive (SRA) database under accession number PRJNA802044.

## Results

### Environmental Parameters

**Figure 2:**
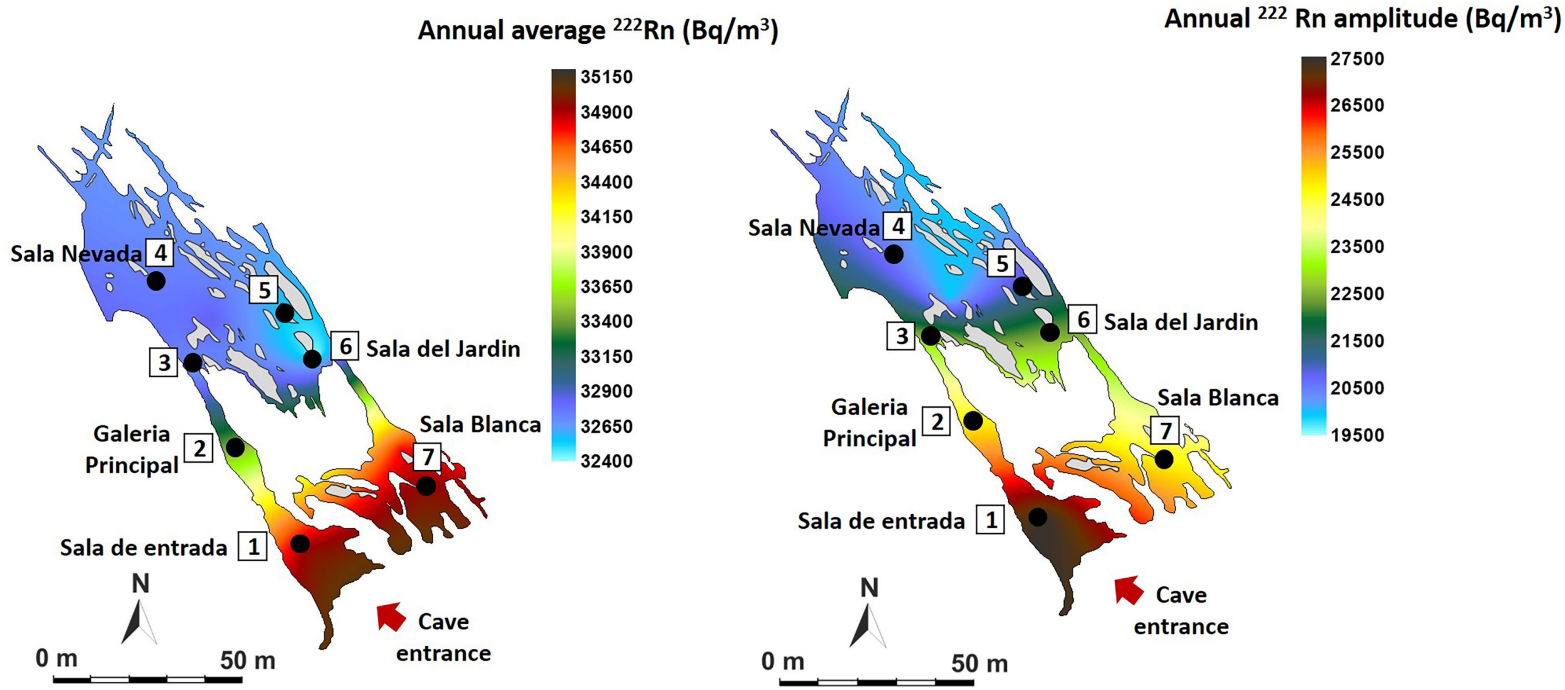
Spatial distribution in Castañar Cave of mean annual radon concentration and maximum amplitude of annual variation expressed in Bq/m^3^.

Figure 2 shows the annual mean values and the annual oscillation ranges of ^222^Rn concentration of the monitored area. The spatio-temporal distribution of tracer gases is directly related to morphology and cave air circulation. The results show that the entire cave has a very high level of natural radiation throughout the year, higher than 30 kBq/m^3^, ranging from 32,440 Bq/m^3^ in the Jardin area (P5-P6) to 34,940 Bq/m^3^ (P1-P7). The highest mean concentrations and the maximum variations (in red tones) are recorded in the area near the entrance (P1) and in Sala Blanca (P7) – Sala Final, the end of the cave. On the contrary, the most stable area throughout the year is Sala Nevada (P4) with an intra-annual variation of 19,942 Bq/m^3^.

**Figure 3:**
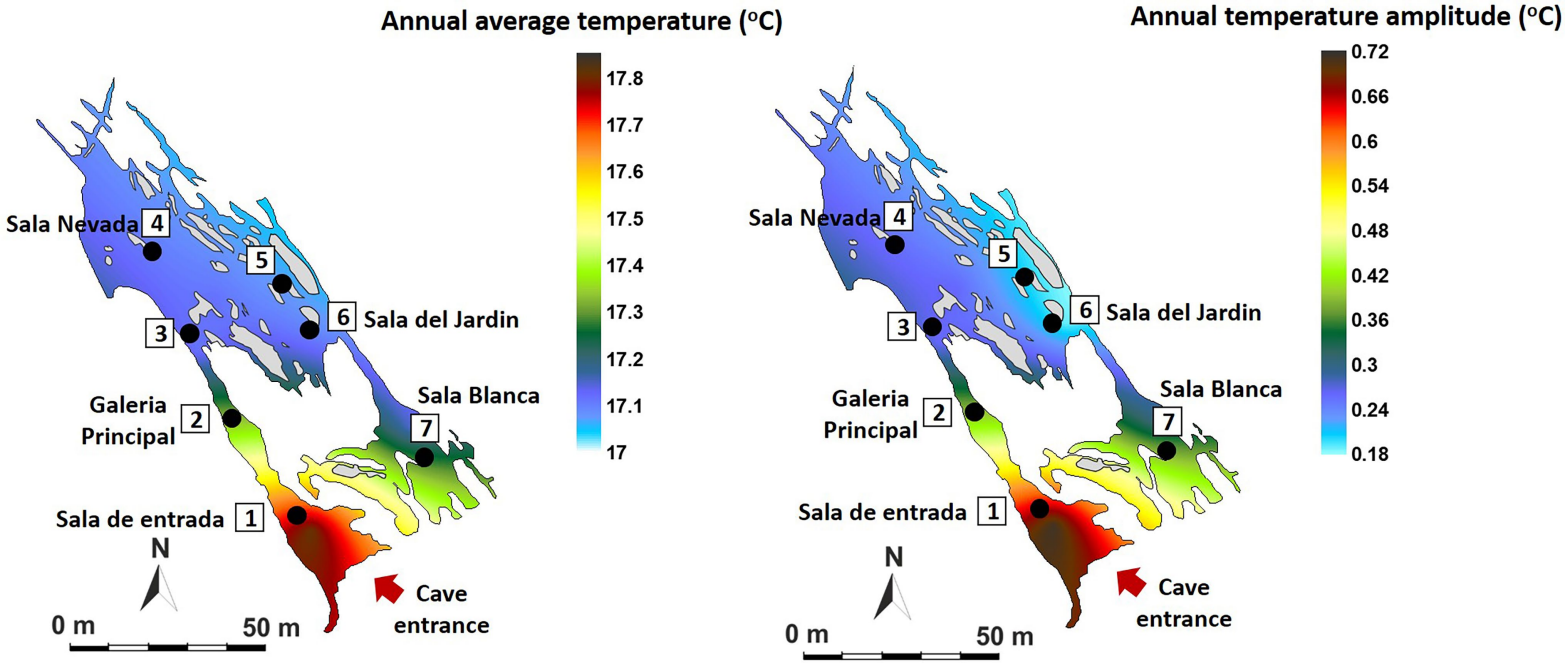
Spatial distribution in Castañar Cave of mean annual air temperature and maximum amplitude of annual variation expressed in degrees centigrade (°C).

In the case of air temperature (Figure 3), the annual mean values range from 17.08°C in the internal zone (P5-P6) to 17.81°C in the areas near the entrance (P1). The data show the high environmental stability of the cave which annual variation ranges below 0.25°C at all measurement points except in the entrance area (P1, 0.71°C of annual amplitude, red tones), where the external direct influence is evident. These data indicate that the sectors close to the entrance show the highest rates of air renewal and consequently of matter and energy exchange with the outside atmosphere. On the contrary, the most stable area with the least energy exchange with the exterior is located in P5-P6 (Sala del Jardin, in light blue tones).

### Fungal Communities in 2020

Six samples from the cave sediments were taken in December 2020 along the touristic trail and one soil sample outside the cave, which were used for molecular studies. Sample P-3 was not studied.

Supplementary Table S1 shows the alpha diversity indices and how fungal diversity decreased in the cave environment when compared to the topsoil. Inside the cave, the lowest fungal diversity was observed in the internal zone (P5-P6), while the highest was found in Sala de Entrada, near the cave entrance. These data indicate that fungal diversity increased with the entry of external organic matter and only a few species survive in the most oligotrophic areas (P5-P6).

**Figure 4:**
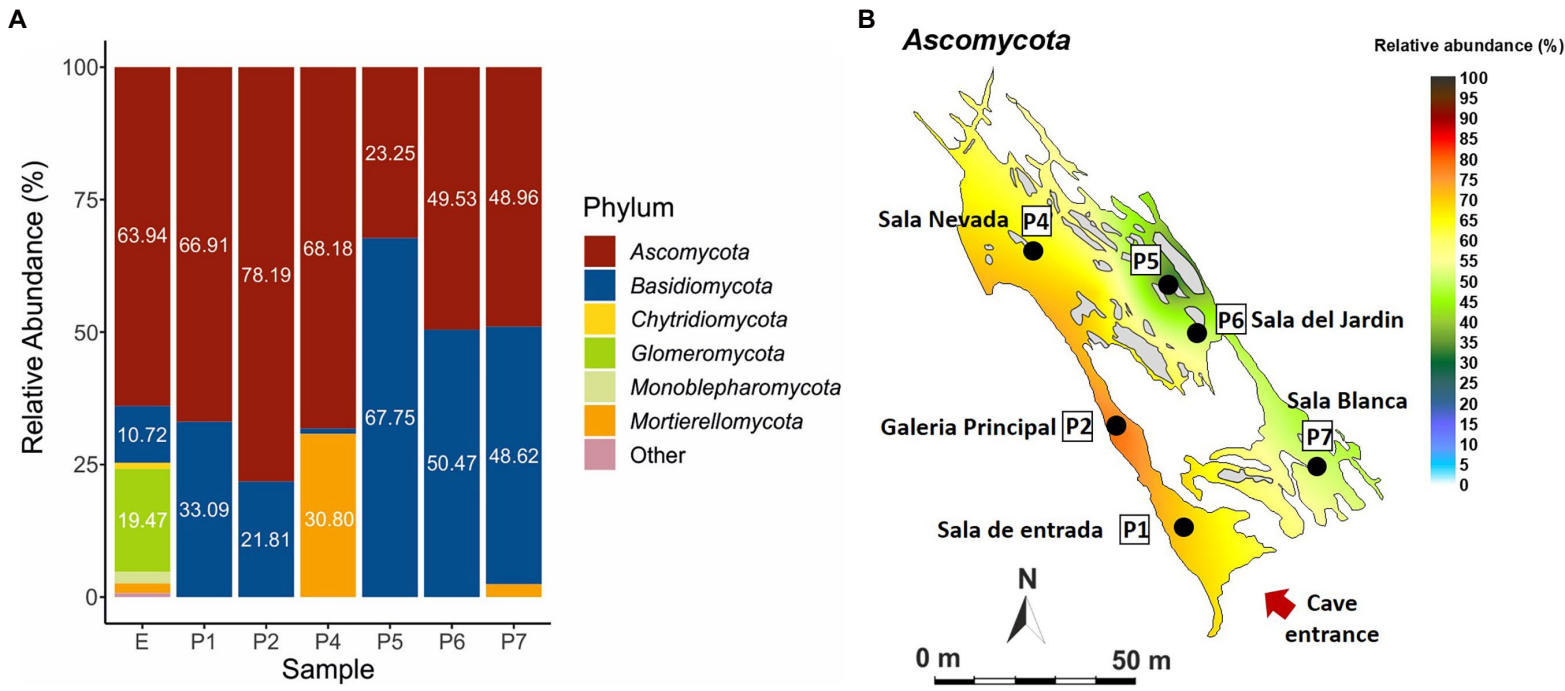
Fungal abundance at phylum level in Castañar Cave, in the December 2020 campaign. **(A)** Barplot illustrating fungal abundance at phylum level in P1-P7, and exterior soil E. Phyla with abundances below 1% are represented as Other. **(B)** Spatial distribution of *Ascomycota* in the December 2020 campaign.

Figure 4 shows phyla distribution in the different samples. In general, there is an uneven distribution between the phyla *Ascomycota* and *Basidiomycota*, with a predominance of ascomycetes in the exterior soil (E) and the first part of the trail from Sala de Entrada (P1) to Sala Nevada (P4), which reversed in the last part of the trail, especially in P5 with a majority of *Basidiomycota*. At the end of the trail, Sala del Jardin (P6) and Sala Blanca (P7) the relative abundances of both phyla were equivalent. Other abundant phyla were *Mortierellomycota* in P4 and *Glomeromycota* in the soil (E). The quantities of other phyla were negligible, below 1%, except for *Monoblepharomycota* that reached 2.1% in the soil (E).

**Figure 5:**
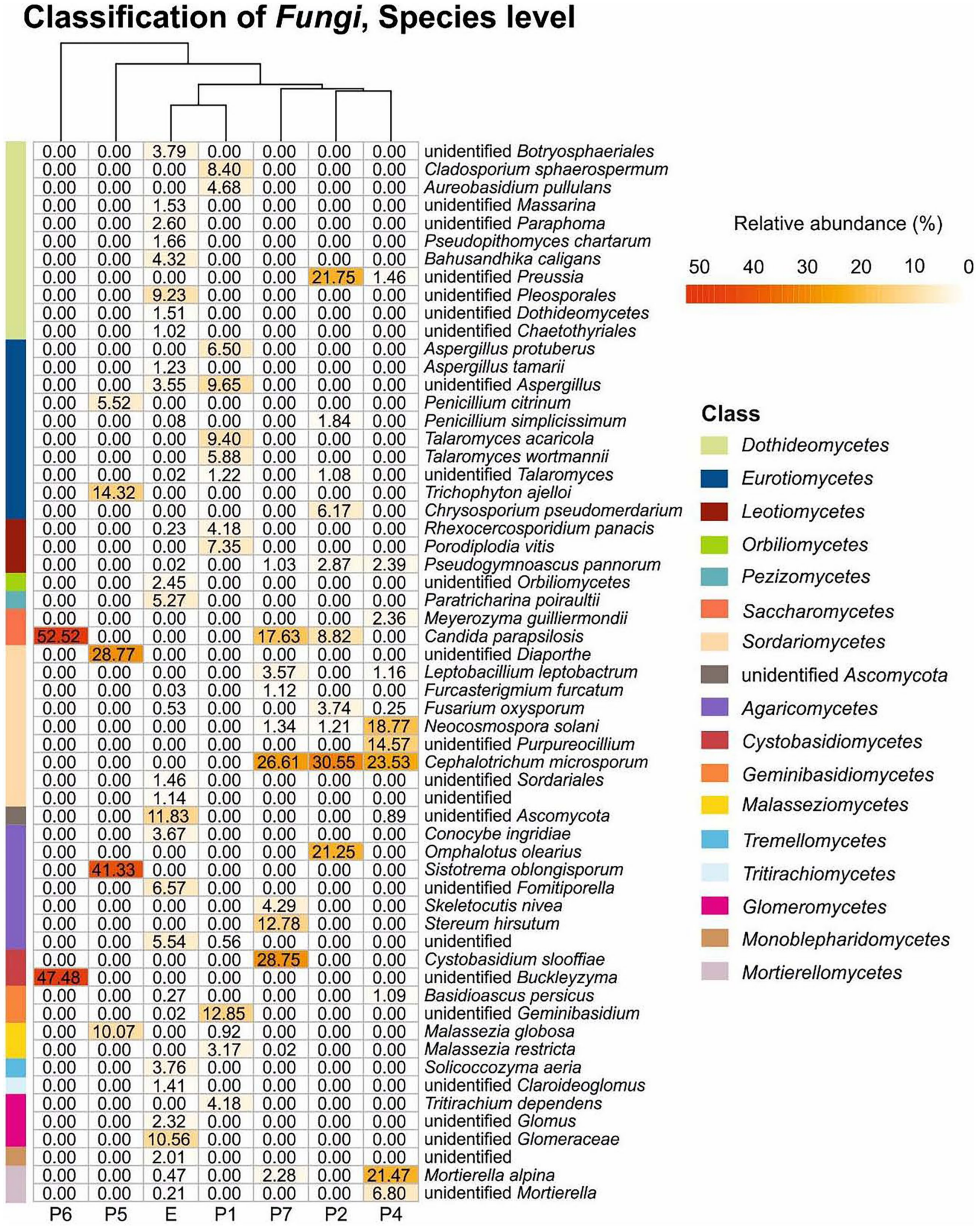
Fungal heat map in Castañar Cave samples. Scales bar show the relative abundance at species level, white squared represents the least abundant species and red the most abundant. Branching patterns on top of each heat map group sediment samples by shared species relative abundance patterns. Bars on the left and the legend on the right indicate the classification at the class level. The species with abundances below 1% are not included.

Figure 5 and Supplementary Table S2 illustrate the relative abundance of major fungi (higher than 1% in at least one sample) at the species level. Thirty five fungi were identified at the species level, while 14 were only at genus level. The most abundant species was *Candida parapsilosis* followed by *Sistotrema oblongisporum*, *Cephalotrichum microsporum*, *Cystobasidium slooffiae*, *Mortierella alpina*, *Omphalotus olearius*, *Neocosmospora solani* (= *Fusarium solani*), *Trichophyton ajelloi*, *Stereum hirsutum*, and *Malassezia globosa*.

Other species identified, although with relative abundance below 5% were *Pseudopithomyces chartarum*, *Bahusandhika caligans*, *Aspergillus tamari*, *Conocybe ingridiae*, and *Solicoccozyma aeria*, which were only found in the soil (E), and *Penicillium simplicissimum, Pseudogymnoascus pannorum, Meyerozyma guilliermondii, Leptobacillium leptobactrum, Fusarium oxysporum, Acremonium furcatum, Skeletocutis nivea*, and *Basidioascus persicus* in different halls.

Among the unidentified members of genera and families were predominant *Buckleyzyma*, *Diaporthe*, *Preussia*, *Purpureocillium*, *Glomeraceae* and *Mortierella*. With relative abundance below 5% were members of the genera *Massarina, Paraphoma*, *Aspergillus*, *Claroideoglomus* and *Glomus* in the soil (E) and *Talaromyces* in the cave.

## Discussion

### Fungi in Castañar Cave 12 Years After a Fungal Outbreak

Only three fungi retrieved from previous surveys in sediments (2008–2009) were found again in the 2020 campaign: *Neocosmospora solani*, *Fusarium oxysporum*, and *Mortierella alpina*. It must be noticed that the comparison was made within isolates and NGS sequences, which could create some bias. Nevertheless, their presence in the cave despite the extensive cleaning of sediments carried after the spillage suggests that these three fungi are resistant and well adapted to the oligotrophic and high radiation conditions of the cave.

The occurrence of *Neocosmospora solani* was detected in three sediment samples (Galeria Principal, Sala Nevada and Sala Blanca) and seems to be a permanent inhabitant of Castañar Cave, present in all samplings since 2008. From a point of view of cave conservation, *Neocosmospora solani* is a dangerous fungus and has been widely reported in relation to cave outbreaks (Dupont et al., 2007; Bastian et al., 2009), and particularly in Castañar Cave (Jurado et al., 2010).

In some caves *Fusarium oxysporum* was associated with *Neocosmospora solani* (Jiang et al., 2017; Popkova and Mazina, 2019), as they are in Castañar Cave. *Fusarium oxysporum* is widely found as a plant pathogen, endophyte, and soil saprobe and was isolated from Chernobyl (Urbaniak et al., 2019).

*Mortierella alpina* and other *Mortierella* species were usually abundant in bat dung samples collected in caves (Degawa and Gams, 2004). *Mortierella* species were reported to be present in all of the 39 samples collected across five habitats, including sediments, weathered rocks, bat guano, drip waters, and air in Heshang Cave, China (Man et al., 2018). In addition to *Fusarium*, *Mortierella* species have been isolated from bat carcasses found in caves (Karunarathna et al., 2020; Nováková, 2021). In Castañar Cave no bats have been reported, but rodents, geckos and their feces were found. We have detected brushite (hydrated calcium phosphate) in the sediments, a mineral from guano-rich caves (Hill and Forti, 1997; Giurgiu and Tamas, 2013). However, brushite is also excreted by rats (Khan, 1998).

Regarding the most abundant taxa (>10%) found in 2020 (Figure 5) 17 taxa stand out. *Candida parapsilosis* was previously isolated from organs and viscera of bats captured in Brazilian urban forests (Ludwig et al., 2023). Other different and abundant *Candida* spp. were isolated from guano and intestinal contents of bats captured in Mexican caves (Ulloa et al., 2006). The occurrence in Castañar Cave is likely associated with the feces of rodents inhabiting the cave.

The genus *Cephalotrichum* seems to be dominant in some caves (Nováková, 2009; Nováková et al., 2018; Vanderwolf et al., 2019). Jiang et al. (2017) isolated 30 strains of *Cephalotrichum* from a Chinese cave, and three were described as new species. *Cephalotrichum microsporum*, a fungus not detected in 2008, was one of the most abundant in the 2020 sampling (Supplementary Table S2; Figure 5), and is a saprophytic fungus often reported from animal dung and soils (Heredia et al., 2018; Supplementary Tables S3, S4). However, its abundance represents a potential risk for the conservation of the cave. As well as *Candida parapsilosis* its presence could be associated with animal dung.

The connection from Sala Nevada to Sala del Jardin (P5) is characterized by the dominance of an unidentified species of *Diaporthe*, followed by *Trichophyton ajelloi*, and *Penicillium citrinum*, among ascomycetes. *Diaporthe* is a genus mainly composed of plant pathogens, responsible for diseases on a wide range of plants, in addition to endophytes and saprobes (Gomes et al., 2013). *Diaporthe* spp. have been isolated from the air in caves from China (Zhang et al., 2017), Brazil (Cunha et al., 2020), Spain (Sanchez-Moral et al., 2021), and from bats in Malaysian caves (Wasti et al., 2020). *Trichophyton ajelloi* (=*Arthroderma uncinatum*) is a geophilic dermatophyte that can cause infections in humans, with a preference for keratin-rich substrates (Zheng et al., 2020). Other *Trichophyton* spp. have been isolated from cave air (Nováková, 2009; Martin-Sanchez et al., 2014; Sanchez-Moral et al., 2021).

Conversely, in the connection from Sala Nevada to Sala del Jardin (P5) the basidiomycetes are composed of *Sistotrema oblongisporum* (41.33%) and *Malassezia globosa* (10.07%). *Sistotrema* spp. have been previously found in mines, always associated with timbers (Eslyn and Lombard, 1983; Held et al., 2020). The presence of these basidiomycetes in the cave could be related with a definite nutrient source, and the most common could be the presence of wood and/or branches fragments. *Malassezia globosa* is a cold-adapted yeast with the ability to survive in extreme conditions (Connell et al., 2014). This species requires lipids for growth and is common in human dandruff and seborrheic dermatitis and may be an indication of human or animal activity (Connell and Staudigel, 2013).

Sala del Jardin (P6) is characterized by the strong relative abundance of two yeasts, *Candida parapsilosis* (*Ascomycota*, *Saccharomycetes*) and an unidentified species of the genus *Buckleyzyma* (*Basidiomycota*, *Cystobasidiomycetes*), both covering 100% of the relative abundance. It has been reported that *Candida parapsilosis* and other yeasts are associated with basidiomycetes (Péter et al., 2017), as we found in Galeria Principal (P2) and Sala Nevada (P7). The genus *Buckleyzyma* comprises species previously placed in the genera *Rhodotorula, Sporobolomyces* and *Bullera* (Wang et al., 2016). Species of *Buckleyzyma* have been isolated from litter (Mašínová et al., 2017), plant leaves and soils (Li et al., 2020), as well as from floor and walls of a winery (Abdo et al., 2020). In most of these environments, *Buckleyzyma* was associated with *Candida, Solicoccozyma, Malassezia, Meyerozyma*, and other yeasts. Species of *Buckleyzyma*, *Candida, Malassezia*, and *Meyerozyma* were found in Castañar Cave and *Solicoccozyma* in the soil outside the cave.

The genus *Preussia* (*Pleosporales, Sporormiaceae*), abundant in the vomit area, Galeria Principal (P2), is widespread on different types of animal dung, and soil, decayed wood, and plant debris. Gonzalez-Menendez et al. (2017) reported that 28 out of 29 species of *Preussia* from the Iberian Peninsula were isolated from dung.

*Omphalotus olearius* (basidiomycetes) and *Chrysosporium pseudomerdarium* (ascomycetes) only appeared in Galeria Principal (P2). The habitat of *Omphalotus olearius* is olive trees (Castro et al., 2011) in accordance with the extensive olive grove under which the cavity is located. This basidiomycete species clearly point to an origin outside the cave and their transport inside, as it was found near the cave entrance. *Chrysosporium pseudomerdarium* was isolated from soils, caves and bat guano (Nagai et al., 1998; Larcher et al., 2003; Saxena et al., 2004). Most species of *Chrysosporium* are keratinolytic (Bohacz, 2017).

A few basidiomycete species appeared exclusively in Sala Blanca (P7): *Cystobasidium slooffiae* (28.75%), *Stereum hirsutum* (12.78%) and *Skeletocutis nivea* (4.29%). *Sistotrema oblongisporum* is a corticioid fungus, as well as *Stereum hirsutum* and *Skeletocutis nivea*. These fungi are wood-rotting species, common in forest on dead branches and logs (Korhonen et al., 2018). Conversely, *Cystobasidium slooffiae*, previous known as *Rhodotorula slooffiae*, was found on bats in Australian caves (Holz et al., 2018). Sala Blanca (P7), at the end of the trail, is rarely visited but the environmental data indicated an external direct influence. The occurrence of these basidiomycetes could be associated to the connection of Sala Blanca with the exterior. Another basidiomycete, an unidentified *Geminibasidium* occurred in Sala de Entrada with relative abundance of 12.85%, but only 0.02% in the exterior soil. The species of this genus have been isolated from forest soils and rhizosphere (Nguyen et al., 2013; Ren et al., 2021).

Other fungi (*Glomeromycota* phylum), only found in the exterior soil, but not in the cave, were the genera *Glomus*, *Claroideoglomus*, and unidentified members of the family *Glomeraceae*, which are arbuscular mycorrhizal fungi (Pagano, 2016). Their obligate association with plants precludes their presence in the cave.

With low relative abundances there are a few interesting fungi in different locations along the cavity: *Pseudogymnoascus pannorum* and *Leptobacillium leptobactrum*.

*Pseudogymnoascus pannorum* is a psychrophilic, keratinolytic fungus (Garzoli et al., 2019), closely related to *Pseudogymnoascus destructans*, the causative agent of white-nose syndrome in bats. This is a soil borne fungus occurring worldwide and in caves. The fungus is common in European and North American caves and is adapted to grow between 4 and 15°C (Out et al., 2016; Vanderwolf et al., 2019; Sanchez-Moral et al., 2021).

The saprotrophic fungus *Leptobacillium leptobactrum* was detected in French and German caves (Bastian et al., 2009; Porca et al., 2011; Burow et al., 2019). In Spanish caves, the abundance of *Leptobacillium leptobactrum* and *Leptobacillium symbioticum* amounted 100% of the fungi isolated from the air in some halls (Dominguez-Moñino et al., 2021). This abundance was suggested to be related to their entomopathogenic activity.

*Meyerozyma guilliermondii* appeared exclusively in Sala Nevada. This is a cold-adapted ascomycete yeast often recorded in Arctic and Antarctic ecosystems (Sannino et al., 2021). *Meyerozyma guilliermondii* occurred in bat guano, in some cases with high isolation frequency (Larcher et al., 2003; Dimkić et al., 2020).

### Spatial Distribution of Fungal Communities in Castañar Cave in 2020

In the 2020 sampling, the distribution of 12 fungal phyla in the soil outside the cave (Figure 4) is similar to that of forest soils. In fact, Kujawska et al. (2021) reported that in forest soils *Ascomycota* was the most abundant phylum, followed by *Basidiomycota*, *Mucoromycota* and *Mortierellomycota*. However, this diversity was greatly reduced inside the cave, with only two phyla (*Ascomycota* and *Basidiomycota*) in samples P1, P2, P5 and P6, and three phyla (*Ascomycota*, *Basidiomycota* and *Mortierellomycota*) in P4 and P7. This loss of diversity can be attributed, among other factors, to the lack of nutrients inside the cave, as opposite to the forest soil. The loss of diversity inside the cave increased in the inner part of the trail (P5-P6), corresponding to the most stable area with the least energy exchange with the exterior.

The uneven distribution of the phyla *Ascomycota* and *Basidiomycota* across the cave is remarkable. Most ascomycetes have been found in areas well connected to the entrance: Sala de Entrada (P1) and Galeria Principal (P2) or in areas with a high impact of tourist visits such as Sala Nevada (P4; Figure 1). Therefore, their presence in the cave is an indirect consequence of the entry of organic matter by three ways: cave animals, human visitors, and airborne spores from outside. The second part of the trail comprises a passage between Sala Nevada and Sala del Jardin (P5), Sala del Jardin (P6), and Sala Blanca (P7; Figure 1), where the relative abundances of basidiomycetes increased, especially in P5. The connection from Sala Nevada to Sala del Jardin (P5) is a very narrow passage that in some way separates both trail sides and this kind of isolation could be responsible of the different niches colonized by ascomycete and basidiomycete species.

It is worth mentioning the abundance of *Mortierellomycota* (30.80%) in Sala Nevada (P4), against *Basidiomycota* (1.01%), contrarily to those found in other halls where *Basidiomycota* ranged from 67.75 to 10.72% (Figure 4). *Mortierellomycota* was only found in Sala Nevada (P4), in addition to Sala Blanca (P7), where reached 2.42%. Most *Mortierellomycota* are saprobic soil-inhabiting fungi and many *Mortierella alpina* strains have been isolated from agricultural soils (Wagner et al., 2011). Other species of *Mortierella* were found in soil samples near the cave. Species of the genus *Mortierella* are common in soils and occur frequently in cave sediments (Nováková, 2009; Zhang et al., 2014; Man et al., 2018; Nováková et al., 2018).

Inside the cave, the fungal diversity was greater in Sala de Entrada (P1), an ecotone area very close to the entrance, where some roots and animals are frequently observed, as well as in P2, P4 and P7, while in the inner areas (P5 and P6) was low (Figure 5). Members of the phyla *Basidiomycota* and *Ascomycota* were found in the soil and Sala de Entrada sediment with high relative abundances. *Glomeromycota* were only found in the soil, as correspond to symbiotic arbuscular mycorrhizal fungi (Pagano, 2016). It was worthy of note the increase of *Basidiomycota* in all cave halls and galleries with respect to the exterior soil, except for Sala Nevada (P4).

It should be noted the high presence of endophytic fungus (*Talaromyces, Porodiplodia vitis, Aureobasidium pullulans*) and fungal species associated with roots and decaying plant material (*Rhexocercosporidium panacis* and *Tritirachium dependens)* in Sala de Entrada (Lu et al., 2014; Vanam et al., 2018; Crous et al., 2019). Therefore, their presence near the entrance was related with roots and the transport of organic matter from outside.

Regarding the ascomycetes, a few fungi were noticeable for their relative abundance: *Candida parapsilosis, Cephalotrichum microsporum*, an unidentified species of the genus *Preussia* and *Neocosmospora solani* were found at different locations along the cavity, while the unidentified species of the genera *Diaporthe* and *Purpureocillium*, as well as *Trichophyton ajelloi* only appeared in Sala Nevada (P4) and/or the connection between Sala Nevada and Sala del Jardin (P5).

### Ecological Traits of Fungi

Castañar Cave is a highly radioactive site with peaks above 50 kBq/m^3^, but also a low-energy cave with oligotrophic conditions (before the vomit spillage) due to the high isolation level from the outdoor environment. These special conditions could affect fungal communities’ development.

Fungi are highly radioresistant when subjected to high doses of ionizing radiation under experimental or accidental conditions (Dadachova and Casadevall, 2008). Chernobyl (Dighton et al., 2008) and the Nevada Test Site (Durrell and Shields, 1960) affected by atmospheric and subterranean nuclear detonations are two reference sites where fungal studies have been carried out. Recently, Fukushima Dai-ichi Nuclear Power Plant was added to the list and it was shown that fungi accumulated high amounts of radiocesium (Fuma et al., 2017). In the literature there are a few papers on the microbial diversity in high ^222^Rn ecosystems (Anitori et al., 2002; Brugger et al., 2005; Weidler et al., 2007).

In the cave sediments the genera *Preussia*, *Aspergillus*, *Acremonium*, *Mortierella* and *Penicillium* were coincident with those found in Chernobyl as well as the species *Pseudogymnoascus pannorum*, *Neocosmospora solani*, *Fusarium oxysporum*, *Penicillium citrinum*, and *Purpureocillium lilacinum* (Zhdanova et al., 1994, 2000; Egorova et al., 2015). However, all these fungi can be considered as cave inhabitants, either in ionized caves or not, which indicates that these species are relatively tolerant to ionizing radiations.

A number of fungi have been studied in relation to the presence or activity in radioactive sites. Some of them have been found in Castañar Cave. Li et al. (2015) reported the isolation of the yeasts *Meyerozyma* (= *Candida*) *guilliermondii* and *Rhodotorula calyptogenae* from a radioactive waste repository. Shuryak et al. (2017) indicated that fungi were particularly resistant to ionizing radiation. These included *Candida parapsilosis* and *Meyerozyma guilliermondii*, in addition to a few *Aspergillus, Acremonium* and *Chrysosporium* species. The two first yeasts were found in the cave as well as different species of the three last fungal genera.

Zhdanova et al. (2000) detected fungal growth on the buildings of Chernobyl and isolated 37 fungi, from which *Penicillium citrinum*, *Pseudogymnoascus pannorum*, *Neocosmospora solani*, and *Fusarium oxysporum* were also found in the cave. All species, except *Pseudogymnoascus pannorum*, were isolated in locations severely contaminated. According to the authors, the annual dose received by these fungi is 10^5^ times the natural background radiation and they noticed that about 80% of the fungi contained melanin.

Blachowicz et al. (2019) investigated the survival of 12 fungi isolated from Chernobyl to UV-C and simulated Mars conditions. Interestingly, among the fungi were represented *Fusarium oxysporum* and different species of *Acremonium*, *Penicillium* and *Aspergillus*.

Gessler et al. (2014) reported that *Purpureocillium lilacinum* strains isolated from Chernobyl areas had a melanin content 2–2.5 times higher than strains from other areas, concluding that radionuclide contamination changed the fungal communities by increasing the amount of melanized fungi, and Egorova et al. (2015) pointed out that this fungus, was used as a bioindicator of the high level of contamination with radionuclides in Chernobyl soils.

Traxler et al. (2021) proved that non-melanized fungi, non-adapted to high ionizing radiation, can survive, grow and spread in the Chernobyl Exclusion Zone soil, with a high level of ionizing radiation. The authors inoculated a model basidiomycete *Schizophyllum commune* into the soil and confirmed that the fungus was present 1 year after inoculation and could cross 1 m distance within 6 months. The test showed that unadapted fungi can thrive in highly contaminated soils where radiation and heavy metals were present.

In light of our data, a high number of non-melanized fungi thrived in the cave sediments but melanized fungi were scarce. This can be explained because cave fungi do not need melanins to protect from UV radiation.

The ecological traits, as well the niches of the most abundant fungal species in Castañar Cave (from P2 to P7) are summarized in Supplementary Tables S3 and S4. Regarding the potential danger to human health, a few species were human opportunistic fungal pathogens: *Candida parapsilosis*, *Meyerozyma guilliermondii*, *Pseudogymnoascus pannorum*, *Leptobacillium leptobactrum*, *Malassezia globosa*. Almost all the fungi in this cave were saprophytes and endophytes and were isolated from caves and soils. As opposed to that observed in other show caves (Dominguez-Moñino et al., 2021), Castañar Cave presented a low occurrence of entomopathogenic fungi: *Leptobacillium leptobactrum* and *Purpureocillium*, both in areas well connected to the outside.

Other different ecological traits were noticed with the identification of psychrophilic (*Candida parapsilosis*, *Malassezia globosa*, *Pseudogymnoascus pannorum*, *Meyerozyma guilliermondii, Buckleyzyma, Penicillium* sp.*, Mortierella, Neocosmospora solani, Fusarium oxysporum, Preussia* sp.), keratinolytic (*Trichophyton ajelloi, Pseudogymnoascus pannorum*, *Chrysosporium pseudomerdarium*, *Penicillium* spp., *Neocosmospora solani*, *Candida parapsilosis*, *Penicillium citrinum*, *Talaromyces*), or coprophilous and/or guano-inhabitant taxa (*Mortierella alpina*, *Preussia* sp.*, Penicillium citrinum*, *Chrysosporium pseudomerdarium*, *Meyerozyma guilliermondii*, *Candida parapsilosis, Talaromyces* spp., *Cephalotrichum microsporum*).

In general, most fungi retrieved are typical soil-borne fungi and widespread in nature (e.g., *Purpureocillium lilacinum*, *Penicillium decumbens*, *Neocosmospora solani*, *Chaetomium globosum*, *Pochonia chlamydosporia*, *Cephalotrichum stemonitis*, *Cephalotrichum microsporum*, *Penicillium citrinum*, *Cladosporium cladosporioides*, *Cladosporium sphaerospermum*, *Aspergillus ustus*, etc.; Domsch et al., 2007).

## Conclusion

The mycobiota of Castañar Cave is defined by the input of organic matter from anthropogenic and animal sources (including dung). It is hypothesized that ecologically distinct niches were created in the cave by the input of diverse types of organic matter, under different environmental conditions and mineral substrata. These niches may account for certain fungal species being present or absent along the cave. The zones well connected to the exterior and Sala Nevada with high impact of tourist visits host copiotrophic fungal species while those thriving in the inner zones occurred under relatively oligotrophic conditions. Under these limited conditions, we observed occasional fungal inhabitants because of the entry of foreign organic matter during visits. The loss of diversity inside the cave increased in the inner part corresponding to the most stable area with the least energy exchange with the exterior.

Regarding persistence, the occurrence of *Neocosmospora solani* in all samplings from 2008 till 2020 is noteworthy since it has been widely reported in relation to cave outbreaks. In addition, this fungus is usually a very abundant soil inhabitant and is able of taking advantage of any disturbance to over develop, as it made during the vomit spillage. An additional attention merits the abundance of *Cephalotrichum microsporum* along the cave. This and other abundant taxa should be controlled.

Fungi previously reported in highly radioactive environments were also found in Castañar Cave, but the effect of high ^222^Rn on these fungi is not conclusive taking into account that the diversity is similar to that found in other caves with relatively low ^222^Rn concentrations.

## Data Availability Statement

The datasets presented in this study can be found in online repositories. The name of the repository and accession number can be found in the article.

## Author Contributions

TM-P, AN, and VJ: samplings and microbial analyses. AF-C, SS-M, and SC: environmental analyses. TM-P and CS-J: writing of the manuscript with support from SS-M and AF-C. SS-M and CS-J: research coordination and funding. All authors have contributed to the scientific discussion of the data and agreed to the submitted version of the manuscript.

## Funding

This research was supported by the Spanish Ministry of Science and Innovation through project PID2019-110603RB-I00 and the collaboration of PID2020-114978GB-I00 project, MCIN/AEI/FEDER, UE/10.13039/501100011033.

## Conflict of Interest

The authors declare that the research was conducted in the absence of any commercial or financial relationships that could be construed as a potential conflict of interest.

## Publisher’s Note

All claims expressed in this article are solely those of the authors and do not necessarily represent those of their affiliated organizations, or those of the publisher, the editors and the reviewers. Any product that may be evaluated in this article, or claim that may be made by its manufacturer, is not guaranteed or endorsed by the publisher.

## Supplementary Material

The Supplementary Material for this article can be found online at: https://www.frontiersin.org/articles/10.3389/fmicb. 2022.869661/full#supplementary-material
