# Supplementary material for: Diversity of Microfungi in a High Radon Cave Ecosystem
Source: Front Microbiol. 2022 Apr 27;13:869661. doi: 10.3389/fmicb.2022.869661 (PMC9093739; doi:10.3389/fmicb.2022.869661)
Supplement: Supplementary file 1 [file Data_Sheet_1.PDF]

Table S1. Raw reads and alpha diversity indices: observed ASVs, Shannon richness and Faith's phylogenetic diversity of fungal communities in December 2020 campaign.

SAMPLE	RAW READS	OBSERVED ASVS	SHANNON	FAITH-PD
E	147167	420	6.96	61.60
P1	14620	45	4.76	10.56
P2	97741	18	2.64	3.39
P4	62713	21	2.61	4.84
P5	37485	8	1.34	2.19
P6	23333	4	0.69	1.61
P7	95589	13	1.87	3.19

Table S2. Relative abundance of fungal species (abundances over 1%) in 2020

Phylum	Class	Order	Family	Genus	Species	E	P1	P2	P4	P5	P6	P7
Ascomycota	*Dothideomycetes*	*Botryosphaeriales*	unidentified	unidentified	unidentified	3 .79	0 .00	0 .00	0 .00	0 .00	0 .00	0 .00
Ascomycota	*Dothideomycetes*	*Capnodiales*	*Cladosporiaceae*	*Cladosporium*	*Cladosporium sphaerospermum*	0 .00	8 .40	0 .00	0 .00	0 .00	0 .00	0 .00
Ascomycota	*Dothideomycetes*	*Dothideales*	*Aureobasidiaceae*	*Aureobasidium*	*Aureobasidium pullulans*	0 .00	4 .68	0 .00	0 .00	0 .00	0 .00	0 .00
Ascomycota	*Dothideomycetes*	*Pleosporales*	*Massarinaceae*	*Massarina*	unidentified	1 .53	0 .00	0 .00	0 .00	0 .00	0 .00	0 .00
Ascomycota	*Dothideomycetes*	*Pleosporales*	*Phaeosphaeriaceae*	*Paraphoma*	unidentified	2 .60	0 .00	0 .00	0 .00	0 .00	0 .00	0 .00
Ascomycota	*Dothideomycetes*	*Pleosporales*	*Pleosporaceae*	*Ulocladium*	*Ulocladium chartarum*	1 .66	0 .00	0 .00	0 .00	0 .00	0 .00	0 .00
Ascomycota	*Dothideomycetes*	*Pleosporales*	*Pleosporales fam Incertae sedis*	*Latorua*	*Latorua caligans*	4 .32	0 .00	0 .00	0 .00	0 .00	0 .00	0 .00
Ascomycota	*Dothideomycetes*	*Pleosporales*	*Sporormiaceae*	*Preussia*	unidentified	0 .00	0 .00	21 .75	1 .46	0 .00	0 .00	0 .00
Ascomycota	*Dothideomycetes*	*Pleosporales*	unidentified	unidentified	unidentified	9 .23	0 .00	0 .00	0 .00	0 .00	0 .00	0 .00
Ascomycota	*Dothideomycetes*	unidentified	unidentified	unidentified	unidentified	1 .51	0 .00	0 .00	0 .00	0 .00	0 .00	0 .00
Ascomycota	*Eurotiomycetes*	*Chaetothyriales*	unidentified	unidentified	unidentified	1 .02	0 .00	0 .00	0 .00	0 .00	0 .00	0 .00
Ascomycota	*Eurotiomycetes*	*Eurotiales*	*Aspergillaceae*	*Aspergillus*	*Aspergillus protuberus*	0 .00	6 .50	0 .00	0 .00	0 .00	0 .00	0 .00

Ascomycota	*Eurotiomycetes*	*Eurotiales*	*Aspergillaceae*	*Aspergillus*	*Aspergillus tamarii*	1 .23	0 .00	0 .00	0 .00	0 .00	0 .00	0 .00
Ascomycota	*Eurotiomycetes*	*Eurotiales*	*Aspergillaceae*	*Aspergillus*	unidentified	3 .55	9 .65	0 .00	0 .00	0 .00	0 .00	0 .00
Ascomycota	*Eurotiomycetes*	*Eurotiales*	*Aspergillaceae*	*Penicillium*	*Penicillium citrinum*	0 .00	0 .00	0 .00	0 .00	5 .52	0 .00	0 .00
Ascomycota	*Eurotiomycetes*	*Eurotiales*	*Aspergillaceae*	*Penicillium*	unidentified	0 .08	0 .00	1 .84	0 .00	0 .00	0 .00	0 .00
Ascomycota	*Eurotiomycetes*	*Eurotiales*	*Trichocomaceae*	*Talaromyces*	*Talaromyces acaricola*	0 .00	9 .40	0 .00	0 .00	0 .00	0 .00	0 .00
Ascomycota	*Eurotiomycetes*	*Eurotiales*	*Trichocomaceae*	*Talaromyces*	*Talaromyces wortmannii*	0 .00	5 .88	0 .00	0 .00	0 .00	0 .00	0 .00
Ascomycota	*Eurotiomycetes*	*Eurotiales*	*Trichocomaceae*	*Talaromyces*	unidentified	0 .02	1 .22	1 .08	0 .00	0 .00	0 .00	0 .00
Ascomycota	*Eurotiomycetes*	*Onygenales*	*Arthrodermataceae*	*Trichophyton*	*Trichophyton ajelloi*	0 .00	0 .00	0 .00	0 .00	14 .32	0 .00	0 .00
Ascomycota	*Eurotiomycetes*	*Onygenales*	*Onygenales fam Incertae sedis*	*Chrysosporium*	*Chrysosporium pseudomerdarium*	0 .00	0 .00	6 .17	0 .00	0 .00	0 .00	0 .00
Ascomycota	*Leotiomycetes*	*Helotiales*	*Helotiales fam Incertae sedis*	*Rhexocercos-poridium*	*Rhexocercospori-dium panacis*	0 .23	4 .18	0 .00	0 .00	0 .00	0 .00	0 .00
Ascomycota	*Leotiomycetes*	*Helotiales*	*Pezizellaceae*	*Porodiplodia*	*Porodiplodia vitis*	0 .00	7 .35	0 .00	0 .00	0 .00	0 .00	0 .00
Ascomycota	*Leotiomycetes*	*Thelebolales*	*Pseudeurotiaceae*	*Pseudogymno-ascus*	unidentified	0 .02	0 .00	2 .87	2 .39	0 .00	0 .00	1 .03
Ascomycota	*Orbiliomycetes*	unidentified	unidentified	unidentified	unidentified	2 .45	0 .00	0 .00	0 .00	0 .00	0 .00	0 .00
Ascomycota	*Pezizomycetes*	*Pezizales*	*Pyronemataceae*	*Paratricharina*	*Paratricharina poiraultii*	5 .27	0 .00	0 .00	0 .00	0 .00	0 .00	0 .00
Ascomycota	*Saccharomycetes*	*Saccharomycetales*	*Debaryomycetaceae*	*Meyerozyma*	*Meyerozyma guilliermondii*	0 .00	0 .00	0 .00	2 .36	0 .00	0 .00	0 .00
Ascomycota	*Saccharomycetes*	*Saccharomycetales*	*Saccharomycetales fam Incertae sedis*	*Candida*	*Candida parapsilosis*	0 .00	0 .00	8 .82	0 .00	0 .00	52 .52	17 .63
Ascomycota	*Sordariomycetes*	*Diaporthales*	*Diaporthaceae*	*Diaporthe*	unidentified	0 .00	0 .00	0 .00	0 .00	28 .77	0 .00	0 .00
Ascomycota	*Sordariomycetes*	*Hypocreales*	*Cordycipitaceae*	*Leptobacillium*	*Leptobacillium leptobactrum*	0 .00	0 .00	0 .00	1 .16	0 .00	0 .00	3 .57
Ascomycota	*Sordariomycetes*	*Hypocreales*	*Hypocreales fam Incertae sedis*	*Acremonium*	*Acremonium furcatum*	0 .03	0 .00	0 .00	0 .00	0 .00	0 .00	1 .12
Ascomycota	*Sordariomycetes*	*Hypocreales*	*Nectriaceae*	*Fusarium*	*Fusarium oxysporum*	0 .53	0 .00	3 .74	0 .25	0 .00	0 .00	0 .00
Ascomycota	*Sordariomycetes*	*Hypocreales*	*Nectriaceae*	*Neocosmospora*	*Neocosmospora solani*	0 .00	0 .00	1 .21	18 .77	0 .00	0 .00	1 .34
Ascomycota	*Sordariomycetes*	*Hypocreales*	*Ophiocordycipita-ceae*	*Purpureocillium*	unidentified	0 .00	0 .00	0 .00	14 .57	0 .00	0 .00	0 .00
Ascomycota	*Sordariomycetes*	*Microascales*	*Microascaceae*	*Cephalotrichum*	*Cephalotrichum microsporum*	0 .00	0 .00	30 .55	23 .53	0 .00	0 .00	26 .61
Ascomycota	*Sordariomycetes*	*Sordariales*	unidentified	unidentified	unidentified	1 .46	0 .00	0 .00	0 .00	0 .00	0 .00	0 .00
Ascomycota	*Sordariomycetes*	unidentified	unidentified	unidentified	unidentified	1 .14	0 .00	0 .00	0 .00	0 .00	0 .00	0 .00

Ascomycota	unidentified	unidentified	unidentified	unidentified	unidentified	11 .83	0 .00	0 .00	0 .89	0 .00	0 .00	0 .00
Basidiomycota	*Agaricomycetes*	*Agaricales*	*Bolbitiaceae*	*Conocybe*	*Conocybe ingridiae*	3 .67	0 .00	0 .00	0 .00	0 .00	0 .00	0 .00
Basidiomycota	*Agaricomycetes*	*Agaricales*	*Omphalotaceae*	*Omphalotus*	*Omphalotus olearius*	0 .00	0 .00	21 .25	0 .00	0 .00	0 .00	0 .00
Basidiomycota	*Agaricomycetes*	*Cantharellales*	*Cantharellales fam Incertae sedis*	*Sistotrema*	*Sistotrema oblongisporum*	0 .00	0 .00	0 .00	0 .00	41 .33	0 .00	0 .00
Basidiomycota	*Agaricomycetes*	*Hymenochaetales*	*Hymenochaetaceae*	*Fomitiporella*	unidentified	0 .00	6 .57	0 .00	0 .00	0 .00	0 .00	0 .00
Basidiomycota	*Agaricomycetes*	*Polyporales*	*Fomitopsidaceae*	*Skeletocutis*	*Skeletocutis diluta*	0 .00	0 .00	0 .00	0 .00	0 .00	0 .00	4 .29
Basidiomycota	*Agaricomycetes*	*Russulales*	*Stereaceae*	*Stereum*	*Stereum hirsutum*	0 .00	0 .00	0 .00	0 .00	0 .00	0 .00	12 .78
Basidiomycota	*Agaricomycetes*	unidentified	unidentified	unidentified	unidentified	0 .56	5 .54	0 .00	0 .00	0 .00	0 .00	0 .00
Basidiomycota	*Cystobasidiomycetes*	*Cystobasidiales*	*Cystobasidiaceae*	*Cystobasidium*	*Cystobasidium slooffiae*	0 .00	0 .00	0 .00	0 .00	0 .00	0 .00	28 .75
Basidiomycota	*Cystobasidiomycetes*	*Cystobasidiomyce-tes ord Incertae sedis*	*Buckleyzymaceae*	*Buckleyzyma*	unidentified	0 .00	0 .00	0 .00	0 .00	0 .00	47 .48	0 .00
Basidiomycota	*Geminibasidiomycetes*	*Geminibasidiales*	*Geminibasidiaceae*	*Basidioascus*	*Basidioascus persicus*	0 .27	0 .00	0 .00	1 .09	0 .00	0 .00	0 .00
Basidiomycota	*Geminibasidiomycetes*	*Geminibasidiales*	*Geminibasidiaceae*	*Geminibasidium*	unidentified	0 .02	12 .85	0 .00	0 .00	0 .00	0 .00	0 .00
Basidiomycota	*Malasseziomycetes*	*Malasseziales*	*Malasseziaceae*	*Malassezia*	*Malassezia globosa*	0 .00	0 .92	0 .00	0 .00	10 .07	0 .00	0 .00
Basidiomycota	*Malasseziomycetes*	*Malasseziales*	*Malasseziaceae*	*Malassezia*	*Malassezia restricta*	0 .00	3 .17	0 .00	0 .00	0 .00	0 .00	0 .02
Basidiomycota	*Tremellomycetes*	*Filobasidiales*	*Piskurozymaceae*	*Solicoccozyma*	*Solicoccozyma aeria*	3 .76	0 .00	0 .00	0 .00	0 .00	0 .00	0 .00
Basidiomycota	*Tritirachiomycetes*	*Tritirachiales*	*Tritirachiaceae*	*Tritirachium*	*Tritirachium dependens*	0 .00	4 .18	0 .00	0 .00	0 .00	0 .00	0 .00
Glomeromycota	*Glomeromycetes*	*Glomerales*	*Claroideoglomera-ceae*	*Claroideoglo-mus*	unidentified	1 .41	0 .00	0 .00	0 .00	0 .00	0 .00	0 .00
Glomeromycota	*Glomeromycetes*	*Glomerales*	*Glomeraceae*	*Glomus*	unidentified	2 .32	0 .00	0 .00	0 .00	0 .00	0 .00	0 .00
Glomeromycota	*Glomeromycetes*	*Glomerales*	*Glomeraceae*	unidentified	unidentified	10 .56	0 .00	0 .00	0 .00	0 .00	0 .00	0 .00
Monoblepharomyco-ta	*Monoblepharidomyce-tes*	unidentified	unidentified	unidentified	unidentified	2 .01	0 .00	0 .00	0 .00	0 .00	0 .00	0 .00
Mortierellomycota	*Mortierellomycetes*	*Mortierellales*	*Mortierellaceae*	*Mortierella*	*Mortierella alpina*	0 .47	0 .00	0 .00	21 .47	0 .00	0 .00	2 .28
Mortierellomycota	*Mortierellomycetes*	*Mortierellales*	*Mortierellaceae*	*Mortierella*	unidentified	0 .21	0 .00	0 .00	6 .80	0 .00	0 .00	0 .00

Table S3. Ecology of fungal species retrieved in 2020

Species	Ecology							References
	Saprobic	Entomo-pathogen	Endophytic	Keratinolytic	Psychrophilic	Human pathogen	Castañar de Ibor location (see Fig. 1)	
Omphalotus olearius	X						P2	(Chliyeh et al. 2014)
Penicillium	X		X	X	X		E, P2	(Chliyeh et al. 2014; El-Gendy 2010; Hassan et al. 2016; Park et al. 2019)
Fusarium oxysporum	X		X		X		E, P2,P4	(Hassan et al. 2016; Jiménez-Fernández et al. 2010)
Neocosmospora solani	X		X	X	X		E, P2,P7	(Anbu et al. 2011; Hassan et al. 2016; Macedo et al. 2017)
Preussia	X				X		P2, P4	(Gupta et al. 2020)
Pseudogymnoascus pannorum	X			X	X	X	P2, P4, P7	(Gianni et al. 2003; Hassan et al. 2016)
Candida parapsilosis				X	X	X	P2, P6, P7	(Buzzini et al. 2012; Trofa et al. 2008; Vermelho et al. 2010)
Meyerozyma guilliermondii			X		X	X	P4	(Buzzini et al. 2012; Chen et al. 2015; Cooper 2011)
Mortierella alpina	X		X		X		E, P4, P7	(Hassan et al. 2016; Wagner et al. 2013; Wani et al. 2017)
Mortierella	X				X		P4, P7	(Hassan et al. 2016; Wagner et al. 2013)
Penicillium citrinum	X		X	X	X		P5	(Anbu et al. 2011; Hassan et al. 2016; Houbraken et al. 2010; Park et al. 2019)
Buckleyzyma	X		X		X		P6	(Buzzini et al. 2018; Mašínová et al. 2017)
Talaromyces	X		X	X			E, P2	(Palem et al. 2015; Tranchida et al. 2016; Yilmaz et al. 2016)
Chrysosporium pseudomerdarium	X		X	X			P2	(Hamayun et al. 2009; Saxena et al. 2004)
Purpureocillium		X	X				P4	(Gong et al. 2017; Nováková 2009)
Cephalotrichum microsporum	X						P2, P4, P7	(Sandoval-Denis et al. 2016)
Leptobacillium leptobactrum	X	X				X	P4, P7	(Okane et al. 2020)
Basidioascus persicus	X						E, P4	(Nasr et al. 2014)
Trichophyton ajelloi				X			P5	(Zheng et al. 2020)
Diaporthe	X		X				P5	(Gomes et al. 2013)
Sistotrema oblongisporum	X						P5	(Christ and Hauerslev 1960)
Malassezia globosa					X	X	P5	(Chandra et al. 2021; Connell and Staudigel 2013)
Acremonium furcatum			X				E, P7	(Maciá-Vicente et al. 2008)
Skeletocutis nivea			X				P7	(Rajchenberg 1983)
Stereum hirsutum	X		X				P7	(Cuevas et al. 2015)

Table S4. Habitat of fungal species retrieved in 2020

Taxonomic assignation	Habitat								References
	Associated olive tree	Soil	Decay wood	Animal dung	Associated with bats	Caves	High radiation	Castañar de Ibor location (see Fig. 1)	
Omphalotus olearius	X		X					P2	(Chliyeh et al. 2014)
Penicillium	X	X		X	X	X	X	E, P2	(Blachowicz et al. 2019; Chliyeh et al. 2014; Dimkić et al. 2020; Nováková 2009; Sanchez-Moral et al. 2021; Tugay et al. 2006)
Fusarium oxysporum	X	X				X	X	E, P2, P4	(Blachowicz et al. 2019; Chliyeh et al. 2014; Jiang et al. 2017; Jiménez-Fernández et al. 2010; Urbaniak et al. 2019)
Neocosmospora solani	X	X				X	X	E, P2, P7	(Chliyeh et al. 2014; Jiang et al. 2017; Macedo et al. 2017)
Preussia		X	X	X				P2, P4	(Gonzalez-Menendez et al. 2017)
Pseudogymnoascus pannorum		X			X	X	X	P2, P4, P7	(Chaturvedi et al. 2018; Zhdanova et al. 2000)
Candida parapsilosis		X		X	X			P2, P6, P7	(Azcón et al. 2010; Ludwig et al. 2021)
Meyerozyma guilliermondii		X		X	X			P4	(Dimkić et al. 2020; Savini et al. 2011)
Mortierella alpina		X		X	X	X		E, P4, P7	(Degawa and Gams 2004; Man et al. 2018)
Mortierella		X		X	X	X		P4, P7	(Degawa and Gams 2004; Man et al. 2018)
Penicillium citrinum		X	X	X	X	X	X	P5	(Dimkić et al. 2020; Houbraken et al. 2010; Nováková 2009; Zhdanova et al. 2000)
Buckleyzyma		X						P6	(Li et al. 2020)
Talaromyces		X	X	X	X	X		E, P2	(Tranchida et al. 2016)
Chrysosporium pseudomerdarium		X			X	X		P2	(Larcher et al. 2003; Saxena et al. 2004)
Purpureocillium		X			X	X	X	P4	(Cunha et al. 2020; Dominguez-Moñino et al. 2021; Egorova et al. 2015)
Cephalotrichum microsporum		X	X	X		X		P2, P4, P7	(Heredia et al. 2018; Sandoval-Denis et al. 2016)
Leptobacillium leptobactrum		X	X			X		P4, P7	(Chavarriaga et al. 2007; Dominguez-Moñino et al. 2021; Kujawska et al. 2021)
Basidioascus persicus		X						E, P4	(Nasr et al. 2014)
Trichophyton ajelloi		X						P5	(Zheng et al. 2020)
Diaporthe		X			X	X		P5	(Gomes et al. 2013; Sanchez-Moral et al. 2021; Wasti et al. 2020)
Sistotrema oblongisporum			X			X		P5	(Christ and Hauerslev 1960; Held et al. 2020)
Malassezia globosa						X		P5	(Connell and Staudigel 2013)
Acremonium furcatum		X						E, P7	(Maciá-Vicente et al. 2008)
Skeletocutis diluta			X					P7	(Rajchenberg 1983)
Stereum hirsutum			X					P7	(Cuevas et al. 2015)
Cystobasidium slooffiae					X	X		P7	(Holz et al. 2018)

References

Anbu, P et al. 2011. “Secretion of Keratinolytic Enzymes and Keratinolysis by *Scopulariopsis brevicaulis* and *Trichophyton mentagrophytes*: Regression Analysis.” *Canadian Journal of MIcrobiology* 52, 1060–1069.

Azcón, R. et al. 2010. “Arbuscular Mycorrhizal Fungi, *Bacillus cereus*, and *Candida parapsilosis* from a Multicontaminated Soil Alleviate Metal Toxicity in Plants.” *Microbial Ecology* 59, 668–77.

Blachowicz, A. et al. 2019. “Proteomic and Metabolomic Characteristics of Extremophilic Fungi Under Simulated Mars Conditions.” *Frontiers in Microbiology* 10, 1013.

Buzzini, P. et al. 2012. “Psychrophilic Yeasts from Worldwide Glacial Habitats: Diversity, Adaptation Strategies and Biotechnological Potential.” *FEMS Microbiology Ecology* 82, 217–241.

Buzzini, P. et al. 2018. “Extremophilic Yeasts: The Toughest Yeasts Around?” *Yeast* 35, 487–97.

Chandra, S.H.V. et al. 2021. “Cutaneous Malassezia: Commensal, Pathogen, or Protector?” *Frontiers in Cellular and Infection Microbiology* 10, 614446

Chaturvedi, V. et al. 2018. “Phenotype Profiling of White-Nose Syndrome Pathogen *Pseudogymnoascus destructans* and Closely-Related *Pseudogymnoascus Pannorum* Reveals Metabolic Differences Underlying Fungal Lifestyles.” *F1000Research* 7, 665.

Chavarriaga, D. et al. 2007. “*Phytophthora cinnamomi* and Other Fine Root Pathogens in North Temperate Pine Forests.” *FEMS Microbiology Letters* 276, 67–74.

Chen, S. et al. 2015. “New Depsidones and Isoindolinones from the Mangrove Endophytic Fungus *Meyerozyma guilliermondii* (HZ-Y2) Isolated from the South China Sea.” *Beilstein Journal of Organic Chemistry* 11, 1187–1193.

Chliyeh, M et al. 2014. “Survey of the Fungal Species Associated to Olive-Tree (*Olea europaea* L.) in Morocco.” *International Journal of Recent Biotechnology* 2, 15–32.

Christ, M.P. and Hauerslev. 1960. “*Sistotrema oblongisporum*.” *Dansk Botanisk Arkiv* 19, 82.

Connell, L. and Staudigel, H. 2013. “Fungal Diversity in a Dark Oligotrophic Volcanic Ecosystem (DOVE) on Mount Erebus, Antarctica.” *Biology* 2, 798–809.

Cooper, C.R. 2011. “Yeasts Pathogenic to Humans.” *The Yeasts* 1, 9–19.

Cuevas, R. et al. 2015. “Extracellular Biosynthesis of Copper and Copper Oxide Nanoparticles by Stereum Hirsutum, a Native White-Rot Fungus from Chilean Forests.” *Journal of Nanomaterials* 2015, 780989.

Cunha, A.O.B. et al. 2020. “Living in the Dark: Bat Caves as Hotspots of Fungal Diversity.” *PLoS ONE* 15, e0243494.

Degawa, Y. and Gams, W. 2004. “A New Species of *Mortierella*, and an Associated Sporangiiferous Mycoparasite in a New Genus, *Nothadelphia*.” *Studies in Mycology* 50, 567–572.

Dimkić, I et al. 2020. “Bat Guano-Dwelling Microbes and Antimicrobial Properties of the Pygidial Gland Secretion of a Troglophilic Ground Beetle against Them.” *Applied Microbiology and Biotechnology* 104, 4109–4126.

Dominguez-Moñino, I. et al. 2021. “Airborne Fungi in Show Caves from Southern Spain.” *Applied Sciences* 11, 5027.

Egorova, A.S. et al. 2015. “Stress Resistance Mechanisms in the Indicator Fungi from Highly Radioactive Chernobyl Zone Sites.” *Microbiology (Russian Federation)* 84, 152–158.

El-Gendy, MM. 2010. “Keratinase Production by Endophytic *Penicillium* spp. Morsy1 under Solid-State Fermentation Using Rice Straw.” *Applied Biochemistry and Biotechnology* 162, 780–94.

Gianni, C. et al. 2003. “Skin Infection Due to *Geomyces pannorum* var. *pannorum*.” *Mycoses* 46, 430–432.

Gomes, R.R. et al. 2013. “*Diaporthe*: A Genus of Endophytic, Saprobic and Plant Pathogenic Fungi.” *Persoonia* 31, 1–41.

Gong, B. et al. 2017. “Endophytic Fungus *Purpureocillium* sp. A5 Protect Mangrove Plant Kandelia Candel under Copper Stress.” *Brazilian Journal of Microbiology* 48, 530–536.

Gonzalez-Menendez, V. et al. 2017. “Biodiversity and Chemotaxonomy of Preussia Isolates from the Iberian Peninsula.” *Mycological Progress* 16, 713–728.

Gupta, P. et al. 2020. “Metagenomic Insights into the Fungal Assemblages of the Northwest Himalayan Cold Desert.” *Extremophiles* 24, 749–758.

Hamayun, M. et al. 2009. “*Chrysosporium pseudomerdarium* Produces Gibberellins and Promotes Plant Growth.” *Journal of Microbiology* 47, 425–430.

Hassan, N. et al. 2016. “Psychrophilic and Psychrotrophic Fungi: A Comprehensive Review.” *Reviews in Environmental Science and Bio/Technology* 15, 147–172.

Held, B.W. et al. 2020. “Diverse Subterranean Fungi of an Underground Iron Ore Mine.” *PLOS ONE* 15, e0234208.

Heredia, G. et al. 2018. “Saprophytic Synnematous Microfungi. New Records and Known Species for Mexico.” *Revista Mexicana de Biodiversidad* 89, 604–618.

Holz, P.H. et al. 2018. “Two Subspecies of Bent-Winged Bats (*Miniopterus orianae bassanii* and *oceanensis*) in

Southern Australia Have Diverse Fungal Skin Flora but Not *Pseudogymnoascus destructans*." *PLoS ONE* 13, e0204282.

Houbraken, J.A.M.P. et al. 2010. "Taxonomy of *Penicillium citrinum* and Related Species." *Fungal Diversity* 44, 117–133.

Jiang, J-R. et al. 2017. "Oligotrophic Fungi from a Carbonate Cave, with Three New Species of *Cephalotrichum*." *Mycology* 8, 164–177.

Jiménez-Fernández, D. et al. 2010. "Identification and Quantification of *Fusarium oxysporum* in Planta and Soil by Means of an Improved Specific and Quantitative PCR Assay." *Applied Soil Ecology* 46, 372–382.

Kujawska, M.B. et al. 2021. "Similarities and Differences among Soil Fungal Assemblages in Managed Forests and Formerly Managed Forest Reserves." *Forests* 12, 353.

Larcher, G. et al. 2003. "Fungal Biota Associated with Bats in Western France." *Journal of Medical Mycology* 13, 29–34.

Li, A.-H. et al. 2020. "Diversity and Phylogeny of Basidiomycetous Yeasts from Plant Leaves and Soil: Proposal of Two New Orders, Three New Families, Eight New Genera and One Hundred and Seven New Species." *Studies in Mycology* 96, 17-140.

Ludwig, L. et al. 2021. "Diversity of Fungi Obtained from Bats Captured in Urban Forest Fragments in Sinop, Mato Grosso, Brazil." *Brazilian Journal of Biology* 83, e247993.

Macedo, R. et al. 2017. "Potential Worldwide Distribution of Fusarium Dry Root Rot in Common Beans Based on the Optimal Environment for Disease Occurrence." *PLOS ONE* 12, e0187770.

Maciá-Vicente, Jose G. et al. 2008. "Fungal Root Endophytes from Natural Vegetation in Mediterranean Environments with Special Reference to *Fusarium* spp." *FEMS Microbiology Ecology* 64, 90–105.

Man, B. et al. 2018. "Diversity of Fungal Communities in Heshang Cave of Central China Revealed by Mycobiome-Sequencing." *Frontiers in Microbiology* 9, 1400.

Mašínová, T. et al. 2017. "Drivers of Yeast Community Composition in the Litter and Soil of a Temperate Forest." *FEMS Microbiology Ecology* 93, fiw223.

Nasr, S. et al. 2014. "*Basidioascus persicus* sp. nov., a Yeast-like Species of the Order *Geminibasidiales* Isolated from Soil." *International Journal of Systematic and Evolutionary Microbiology* 64, 3046–3052.

Nováková, A. 2009. "Microscopic Fungi Isolated from the Domica Cave System (Slovak Karst National Park, Slovakia). A Review." *International Journal of Speleology* 38, 71–82.

Okane, I. et al. 2020. "A New Species of *Leptobacillim, L. symbioticum*, Isolated from Mites and Sori of Soybean Rust." *Mycoscience* 61, 165–171.

Palem, P.P.C. et al. 2015. "An Endophytic Fungus, *Talaromyces radicus*, Isolated from *Catharanthus roseus*, Produces Vincristine and Vinblastine, Which Induce Apoptotic Cell Death." *PLOS ONE* 10, e0144476.

Park, M.S. et al. 2019. "The Diversity and Ecological Roles of *Penicillium* in Intertidal Zones." *Scientific Reports* 9, 13540.

Rajchenberg, M. 1983. "New South American Resupinate Polypores." *Mycotaxon* 16, 500–506.

Sanchez-Moral, S. et al. 2021. "Environment-Driven Control of Fungi in Subterranean Ecosystems: The Case of La Garma Cave (Northern Spain)." *International Microbiology* 24, 573–591.

Sandoval-Denis, M. et al. 2016. "Phylogeny and Taxonomic Revision of Microascaceae with Emphasis on Synnematous Fungi." *Studies in Mycology* 83, 193–233.

Savini, V. et al. 2011. "What Do We Know about Candida Guilliermondii? A Voyage throughout Past and Current Literature about This Emerging Yeast." *Mycoses* 54, 434–441.

Saxena, P. et al. 2004. "Diversity of Keratinophilic Mycoflora in the Soil of Agra (India)." *Folia Microbiologica* 49, 430–434.

Tranchida, M.C. et al. 2016. "First Record of *Talaromyces udagawae* in Soil Related to Decomposing Human Remains in Argentina." *Revista Argentina de Microbiología* 48, 86–90.

Trofa, D. et al. 2008. "*Candida parapsilosis*, an Emerging Fungal Pathogen." *Clinical Microbiology Reviews* 21, 606–625.

Tugay, T. et al. 2006. "The Influence of Ionizing Radiation on Spore Germination and Emergent Hyphal Growth Response Reactions of Microfungi." *Mycologia* 98, 521–527.

Urbaniak, C. et al. 2019. "Genomic Characterization and Virulence Potential of Two *Fusarium oxysporum* Isolates Cultured from the International Space Station." *mSystems* 4, e00345-18.

Vermelho, A.B. et al. 2010. "Identification of a *Candida parapsilosis* Strain Producing Extracellular Serine Peptidase with Keratinolytic Activity." *Mycopathologia* 169, 57–65.

Wagner, L. et al. 2013. "A Comprehensive Molecular Phylogeny of the *Mortierellales* (*Mortierellomycotina*) Based on Nuclear Ribosomal DNA." *Persoonia* 30, 77–93.

Wani, Z.A. et al. 2017. "*Mortierella alpina* CS10E4, an Oleaginous Fungal Endophyte of *Crocus sativus* L. Enhances Apocarotenoid Biosynthesis and Stress Tolerance in the Host Plant." *Scientific Reports* 7, 8598.

Wasti, I.G. et al. 2020. "Fungi from dead arthropods and bats of Gomantong Cave, northern Borneo, Sabah (Malaysia)." *Journal of Cave and Karst Studies* 82, 261–275.

Yilmaz, N. et al. 2016. "Four Novel *Talaromyces* Species Isolated from Leaf Litter from Colombian Amazon Rain Forests." *Mycological Progress* 15, 1041–1056.

Zhdanova, N.N. et al. 2000. "Fungi from Chernobyl: Mycobiota of the Inner Regions of the Containment Structures of the Damaged Nuclear Reactor." *Mycological Research* 104, 1421–26.

Zheng, H. et al. 2020. "Assembly and Analysis of the Whole Genome of *Arthroderma uncinatum* Strain T10, Compared with *Microsporum canis* and *Trichophyton rubrum*." *Mycoses* 63, 683–693.
